# Oral hygiene practices and their socio-demographic correlates among Nepalese adult: evidence from non communicable diseases risk factors STEPS survey Nepal 2013

**DOI:** 10.1186/s12903-016-0294-9

**Published:** 2016-09-29

**Authors:** Pushpa Thapa, Krishna Kumar Aryal, Suresh Mehata, Abhinav Vaidya, Bijay Kumar Jha, Meghnath Dhimal, Shaili Pradhan, Purushottam Dhakal, Arpana Pandit, Achyut Raj Pandey, Bihungum Bista, Ava Upadhyay Pokhrel, Khem Bahadur Karki

**Affiliations:** 1 Nepal Health Research Council, Ramshahpath, Kathmandu, 44600 Nepal; 2 Nepal Health Sector Support Program, Ministry of Health, Kathmandu, 44600 Nepal; 3 Kathmandu Medical College, Sinamangal, Kathmandu, 44600 Nepal; 4 National Academy of Medical Sciences, Kathmandu, 44600 Nepal; 5 Nobel Medical College and Teaching Hospital, Morang, 56600 Nepal

**Keywords:** Non-communicable diseases risk factors, Nepal, Oral health, Oral hygiene practices, Socio-demographic factors, STEPS survey

## Abstract

**Background:**

Oral diseases remain a significant public health problem in Nepal, as do oral health behaviours. Socio-demographic factors play a crucial role in driving oral hygiene practices. This study aims to identify oral hygiene practices and associated socio-demographic factors in Nepalese population.

**Methods:**

This descriptive, cross-sectional study recruited 4200 adults (15–69 years) through multistage cluster sampling. Data obtained from the WHO NCD STEPS instrument version 2.2 were analysed in STATA 13.0 using complex sample weighted analysis.

**Results:**

Prevalence of cleaning teeth at least once a day was 94.9 % (95 % CI: 93.7–95.9), while that of cleaning teeth at least twice a day was 9.9 % (95 % CI: 8.2–11.9). Use of fluoridated toothpaste was seen among 71.4 % (95 % CI: 67.9–74.7) respondents. A 3.9 % (95 % CI: 3.1–5.0) made a dental visit in the last 6 months.

The 45–69 years age group had lesser odds of cleaning teeth at least once a day (AOR: 0.4; 95 % CI: 0.2–0.8), in comparison to 15–29 years age group. Women had greater odds of cleaning teeth at least twice a day (AOR: 1.7; 95 % CI: 1.1–2.4) and having visited a dentist in the last 6 months (AOR: 2.2; 95 % CI: 1.2–3.8) compared to men. With reference to rural residents, urban population had higher odds of using fluoridated toothpaste (AOR: 2.3; 95 % CI: 1.4–3.4) and making a dental visit within the last 6 months (AOR: 1.9; 95 % CI:1.1–3.6). Inhabitants of the Terai had five-fold (AOR: 4.9; 95 % CI: 3.1–7.8) greater odds of cleaning teeth once per day than did hill residents. Those with higher education had greater odds than non-formal education holders of cleaning teeth at least once a day (AOR: 9.0; 95 % CI: 2.9–27.7), cleaning teeth at least twice a day (AOR: 5.6; 95 % CI: 2.9–10.6), using fluoridated toothpaste (AOR: 13.9; 95 % CI: 8.4–23.1), and having visited a dentist in the last 6 months (AOR: 2.8; 95 % CI: 1.4–5.4).

**Conclusions:**

Cleaning teeth at least once a day is widely prevalent in Nepal and a substantial number of population use fluoridated toothpaste. However, cleaning teeth twice a day and visiting a dentist is less common. Being women, Terai residents, urban residents, and educated were significantly associated with oral hygiene practices assessed in this study.

**Electronic supplementary material:**

The online version of this article (doi:10.1186/s12903-016-0294-9) contains supplementary material, which is available to authorized users.

## Background

Oral diseases are a major public health concern globally [1], and profoundly impact the well being and daily social lives of people [2]. Worldwide, 3.9 billion people are affected by oral diseases [3]. Disadvantaged and poor populations are the most vulnerable worldwide [1].

Poor oral hygiene is recognised as one of the greatest risk factors for oral diseases [1]. Evidence shows that brushing teeth at least twice a day and making dental visits help to counter oral health problems [4]. Oral hygiene practices are in turn influenced by socio-demographic environment [1, 5]. Higher education [6, 7], socioeconomic status [8] have been found to contribute to oral hygiene habits and utilisation of oral health services.

Despite being highly preventable, a substantial proportion of the Nepalese population experiences oral health problems [9], especially poor and marginalized individuals [10]. One-third of Nepalese adults have been found to have self-reported dental caries [11]. Modernisation and changes in dietary patterns have all contributed to the poor oral health status in Nepal [10].

There have been very few studies documenting oral hygiene practices and related socio-demographic factors in the context of Nepal. Being one of the poorest countries [12], the cost of dental services is prohibitive for the majority of the Nepalese populace [10]. Against this background, the identification of oral health practices and related socio-demographic factors in Nepal is crucial to the design and implementation of appropriate oral health promotion interventions. The present study thus assessed oral hygiene practices and its socio-demographic correlates among the adult Nepalese population.

## Methods

This study is a part of a national STEPS survey on non-communicable diseases (NCD) risk factors which had World Health Organization (WHO) recommended oral health module to identify risk factors on oral health.

### Study design and population

This was a nationally representative descriptive, cross-sectional study conducted from January to June 2013, assigning adult men and women aged 15–69 years as the study population. Only those who had been living at their current place of residence for at least 6 months were included in the study. The study excluded too frail and mentally ill who were unable to provide responses. We also excluded people with physical disability as anthropometric measurements were also one of the major variables to be measured for the main study even though this paper does not make use of that particular variable. Further, we excluded those with primary places of residence in military bases or group dwellings; and those residing in hospitals, prisons, nursing homes or other institutions.

### Sampling method

Multistage cluster sampling was employed and carried out at three stages as explained elsewhere [13]. Sample size of 4,200 was calculated using the prevalence of low fruit and vegetables consumption (61.9 %) from the NCD Risk Factors Survey 2008 [14] as baseline level of indicator, with 95 % confidence level, 5 % of absolute error, 1.5 design effect and 80 % expected response.

Ilakas (sub district units comprising of village development committees (VDCs) and/or whole or part of municipalities) made the primary sampling units (PSUs). Individual wards (lowest administrative division under the VDCs and municipalities) were the secondary sampling units (SSUs) and individual households made the tertiary sampling units (TSUs). PSUs and SSUs were selected using probability proportionate to size (PPS) sampling technique and TSUs were selected using systematic random sampling. One eligible respondent (15–69 years) from each selected household was selected using the Kish method and approached for inclusion in the study [15]. The response rate was 98.6 % (4,143 adults participated in STEP I of the survey which included measurement of behavioural risk factors including that of oral health).

### Data collection tools and technique

The survey was conducted using the WHO NCD STEPS instrument version 2.2 [16], which prescribes three steps for measuring NCD risk factors. STEP I measures behavioural risk factors, STEP II covers physical measurements, and STEP III measures biological risk factors. Oral hygiene-related behavior is covered in STEP I as one of the optional modules, of which we used all the questions prescribed in the stepwise approach to surveillance [16]. We report four individual oral health behaviors as assessed in the NCD Risk Factors STEPS Survey Nepal 2013, which are: cleaning teeth at least once a day, cleaning teeth at least twice a day, using fluoridated tooth paste, and having visited a dentist in the last 6 months.

A week-long training was conducted for enumerators and field supervisors. All the enumerators came from a health background, and supervisors held a bachelor degree in public health with substantial experience in research work. Focused training agendas were: interview techniques, sampling process, household and individual selection, use of different kinds of templates and forms in the survey, use and care of personal digital assistants (PDAs), and detailed explanations of the questionnaire and physical measurement techniques. Supervisors were also trained on downloading data from PDAs and handling issues with PDAs. The STEPS instrument was translated into Nepali and validated through a pilot study carried out in Kirtipur Municipality, Kathmandu, among 20 households. Necessary changes were made to the questionnaire; the revised version was then endorsed by a Steering Committee prior to field use. Enumerators carried out data collection using PDAs. Frequent monitoring and supervision was carried out from the central level during data collection.

### Major variables in the study

#### Education level

Education level was defined based on the literacy status of the respondents. Respondents with non-formal education were those who did not attend any formal education. Other levels were categorized as per the number of school years: primary education (5 years of schooling completed), secondary education (10 years of schooling completed), and higher secondary or above (more than or equal to 12 years of schooling completed).

#### Place of residence

Based on the central bureau of statistics, we categorized village development committees (VDCs) as rural and municipalities as urban in this study.

#### Ecological belt

Nepal is geographically divided into three belts north to south based on altitude. Mountains, hills and the Terai are three ecological belts where mountains refers to the high mountain region, hills refers to adjacent region in a lower altitudinal belt while the Terai refers to the lowest terrain in the country ranging from 70 to 700 m above the sea level.

### Data analysis

Data collected in PDAs were exported to Microsoft Excel 2007, cleaned with SPSS 16.0, and analysed with STATA 13.0 to carry out complex sample weighted analysis. To measure the association between oral hygiene practices and socio-demographic factors, adjusted odds ratios (AOR) were estimated through logistic regression models. We adjusted age, gender, ecological belt, place of residence, education level and marital status in the logistic regression models. Weighting was done so as to be able to generalise the findings to adults aged 15–69 years across the country. As the study used multistage sampling, weight was calculated by using the probability of selections of PSUs, SSUs, TSUs and individual respondents. Firstly, the probabilities of selections at the individual level were calculated and multiplied to get a final probability. The inverse of the selection probability was then used as a sample weight.

## Results

A total of 4,143 adults participated in STEP I, which contained the module on oral health. We report the results for 4143 observations for all the variables except marital status where we had two missing responses.

### Oral hygiene practices

Of the total, almost 95 % reported cleaning teeth at least once a day, while around 10 % reported cleaning teeth twice a day. Among the total population, 71 % used fluoridated toothpaste and only 4 % visited dentist in the last 6 months (Table 1).

**Table 1:** Distribution of oral hygiene practices by socio-demographic characteristics

Characteristics	N (un-weighted %)	Cleaning teeth at least once a day	Cleaning teeth at least twice a day	Use of fluoridated toothpaste	Visited a dentist within the last 6 months
		% (95 % CI)	% (95 % CI)	% (95 % CI)	% (95 % CI)
Age Group (Years)					
15–29	972 (23.5)	97.9(96.8–98.7)	13.1(10.4–16.3)	79.3(75.1–82.9)	2.8(1.8–4.4)
30–44	1558 (37.6)	94.8(92.9–96.2)	8.2(6.3–10.5)	69.1(64.6–73.2)	4.0(2.8–5.6)
45–69	1613 (38.9)	89.6(87.1–91.6)	6.2(4.6–8.2)	57.6(52.6–62.4)	6.1(4.5–7.6)
Gender					
Men	1336 (32.2)	95.7(94.3–96.8)	9.6(7.3–12.4)	75.7(71.6–79.9)	3.1(1.8–4.8)
Women	2807 (67.8)	94.1(92.4–95.4)	10.3(8.2–12.3)	66.1(62.3–69.7)	4.8(3.8–6.2)
Ecological Belt					
Mountain	300 (7.1)	84.3(72.6–91.5)	4.2(0.6–2.3)	70.7(59.1–80.2)	4.6(1.5–12.6)
Hill	1800 (42.9)	92.60(90.3–94.3)	10.2(7.8–13.1)	75.2(70.4–79.6)	3.9(2.5–6.0)
Terai	2100 (50.0)	98.2(97.4–98.7)	11.0(7.9–13.7)	67.0(61.3–72.3)	3.9(2.9–5.3)
Place of Residence					
Rural	3422 (18.5)	94.3(92.9–95.4)	8.5(6.8–10.6)	67.3(63.3–71.1)	3.3(2.4–4.5)
Urban	778 (81.5)	97.2(94.6–98.6)	16.0(11.2–22.1)	85.4(79.9–89.8)	6.7(4.4–10.0)
Education Level					
Non Formal	1851 (44.7)	88.3(85.3–90.7)	4.1(2.8–5.4)	44.6(40.4–49.1)	3.9(2.9–5.2)
Primary	102 (24.6)	95.8(94.1–97.1)	7.0(4.6–10.4)	71.6(66.5–76.1)	4.0(2.6–6.0)
Secondary	773 (18.7)	98.9(97.9–99.5)	13.5(10.5–17.2)	85.3(80.8–88.9)	2.9(1.9–4.4)
Higher Secondary or above	498 (12.0)	98.8(96.1–99.6)	19.0(14.4–24.6)	92.8(89.6–95.1)	5.3(3.2–8.7)
Marital Status^a^					
Never Married	336 (8.1)	98.2(95.8–99.3)	16.2(11.9–21.6)	85.2(78.7–90.0)	3.5(1.9–6.5)
Currently Married	3568 (86.2)	94.7(93.4–95.8)	8.7(7.1–10.6)	68.2(64.6–71.6)	4.0(3.1–5.2)
Divorced/Widowed/Separated	237 (5.7)	79.1(71.5–85.2)	4.6(2.4–7.6)	49.9(42.1–57.8)	5.4(2.8–9.0)
Total	4143	94.9(93.8–96.0)	9.9(8.2–11.9)	71.4(67.9–74.7)	3.9(3.1–5.0)

^a^Two refused response

### Distribution of oral hygiene practices across various socio-demographic strata

Around 98 % of the people aged 15–29 years cleaned teeth at least once a day, while prevalence of cleaning teeth at least twice a day was also the highest among the same age group. A similar proportion of men and women cleaned teeth at least once a day and at least twice a day. Nearly, all people from the Terai, from urban areas, with secondary education or above and never married cleaned teeth at least once a day. Prevalence of cleaning teeth at least twice a day was found 16 % among urban inhabitants, 19 % among higher secondary or above education holders and 16 % among never married individuals. Use of fluoridated toothpaste was most common among the youngest age group - 15–29 year olds (79 %), urban dwellers (85 %), higher secondary or above education holders (93 %), and never married individuals (85 %) (Table 1).

### Socio-demographic correlates of oral hygiene practices

#### Cleaning teeth at least once a day

The odds of cleaning teeth at least once a day was five times higher among people from the Terai compared to the hill residents. The odds of cleaning teeth at least once a day increased with increased level of education (Table 2).

**Table 2:** Socio-demographic correlates of oral hygiene practices

	Cleaning teeth at least once a day		Cleaning teeth at least twice a day		Use of fluoridated toothpaste		Visited a dentist within the last 6 months	
	Crude OR	Adjusted OR	Crude OR	Adjusted OR	Crude OR	Adjusted OR	Crude OR	Adjusted OR
Age Group (Years)								
15–29	1	1	1	1	1	1	1	1
30–44	0.38(0.22–0.63)	0.6(0.4–1.1)	0.6(0.4–0.8)	0.9(0.6–1.3)	0.6(0.5–0.7)	1.1(0.8–1.4)	1.4(0.9–2.4)	2.0(1.0–3.8)
45–69	0.17(0.10–0.29)	0.4(0.2–0.8)	0.4(0.3–0.6)	0.9(0.6–1.4)	0.4(0.3–0.5)	0.9(0.7–1.2)	2.2(1.3–3.5)	3.6(1.8–7.1)
Gender								
Men	1	1	1	1	1	1	1	1
Women	0.7(0.4–0.9)	1.3(0.9–1.91)	1.1(0.8–1.5)	1.7(1.1–2.4)	0.6(0.5–0.8)	1.2(0.9–1.5)	1.6(0.9–2.7)	2.2(1.2–3.8)
Ecological Belt								
Hill	1	1	1	1	1	1	1	1
Mountain	0.4(0.2–0.8)	0.5(0.2–1.1)	0.4(0.6–2.4)	0.5(0.8–3.3)	0.8(0.5–1.4)	1.1(0.7–1.8)	1.1(0.4–3.5)	1.6(0.5–5.2)
Terai	4.4(2.7–7.0)	4.9(3.1–7.8)	1.0(0.7–1.6)	1.1(0.7–1.7)	0.7(0.5–0.9)	0.7(0.5–0.9)	1.0(0.6–1.7)	1.2(0.6–2.1)
Residence								
Rural	1	1	1	1	1	1	1	1
Urban	2.1(1.0–4.5)	1.5(0.7–3.1)	2.0(1.3–3.2)	1.6(0.9–2.6)	2.8(1.9–4.4)	2.3(1.5–3.4)	2.1(1.2–3.5)	1.9(1.1–3.6)
Education Level								
Non Formal	1	1	1	1	1	1	1	1
Primary	3.1(2.0–4.6)	3.0(2.0–4.4)	1.8(1.2–2.9)	2.0(1.2–3.1)	3.1(2.4–3.9)	3.0(2.4–4.0)	1.0(0.6–1.7)	1.6(1.0–2.6)
Secondary	13.0(6.0–28.1)	10.3(4.4–24.0)	3.8(2.6–5.7)	4.0(2.4–6.3)	7.2(5.3–9.9)	7.2(5.1–10.3)	0.7(0.5–1.1)	1.4(0.8–2.4)
Higher	11.2(3.1–40.2)	9.0(2.9–27.7)	5.7(3.7–9.0)	5.6(2.9–10.6)	16.0(10.6–24.3)	14.3(8.7–23.7)	1.3(0.8–2.4)	2.8(1.4–5.4)
Marital Status								
Never Married	1	1	1	1	1	1	1	1
Currently Married	0.3(0.1–0.8)	1.1(0.4–2.6)	0.5(0.6–2.4)	0.8(0.4–1.2)	0.4(0.2–0.6)	0.9(0.5–1.5)	1.1(0.6–2.2)	0.7(0.3–1.7)
Divorced/Widowed/Separated	0.1(0.0–0.2)	0.4(0.2–1.1)	0.2(0.7–1.6)	0.6(0.2–1.3)	0.2(0.1–0.3)	0.8(0.4–1.5)	1.5(0.6–3.5)	0.7(0.2–2.0)

#### Cleaning teeth at least twice a day

Women had 1.7 times higher odds of cleaning teeth at least twice a day than men. The odds of cleaning teeth at least twice a day rose with educational level similar to cleaning teeth at least once a day. Compared to those with non-formal education, odds of cleaning teeth at least twice a day was 2 times higher among primary education holders, four times higher among secondary education holders, and 5.6 times higher among higher secondary and above education holders (Table 2).

#### Use of fluoridated toothpaste

Residents of the Terai had lesser odds of using fluoridated toothpaste than hill-dwelling residents (AOR: 0.7; 95 % CI: 0.4–0.9). Urban inhabitants had higher odds of using fluoridated toothpaste use (AOR: 2.3; 95 % CI: 1.5–3.4) than rural residents. People with higher secondary education and above had greater odds (AOR: 14.3; 95 % CI: 8.7–23.7) of using fluoridated toothpaste, compared to those with non-formal education (Table 2).

#### Visited a dentist within the last 6 months

People in the oldest age group, 45–69 years, had higher odds (AOR: 3.6; 95 % CI: 1.8–7.1) of having sought dental services in the last 6 months than those in the youngest age group, 15–29 years. Likewise, women had higher odds of seeking dental services (AOR: 2.2; 95 % CI: 1.2–3.8) than men and the urban population had greater odds (AOR: 1.9, 95 % CI: 1.1–3.6) than the rural. Higher secondary or above education holders had 3 times higher odds of utilizing dental services compared to those with non-formal education (Table 2).

## Discussion

This study provides a snapshot of oral hygiene practices and their socio-demographic correlates among the adult population of Nepal. As is strongly fostered by the National Oral Health Policy 2014 on the execution of oral health related surveys [10], the findings yielded by this countrywide study provide a strong basis from which to commence oral health promotion interventions.

We found a mixed result of the selected oral hygiene practices that are included in this paper with a substantial proportion of people cleaning teeth at least once a day and using fluoridated toothpaste. On the other hand, only a small proportion cleaned teeth at least twice a day. Oral hygiene practices are directly linked with several oral and periodontal disorders [4, 17] and are also linked to some chronic conditions [18, 19]. This hints the need of strategies to promote oral hygiene practices for preventing oral diseases as well as some other chronic conditions. Integrating oral diseases into prevention and control of NCDs could be beneficial as they share the common risk factors such as tobacco use, alcohol consumption, and unhealthy diets high in sugar [2]. Only one in ten respondents was found to be cleaning teeth twice a day, a marked differences with the findings of similar studies done in Denmark (68.0 %) and Kuwait (62.0 %) [4, 20]. While this figure was 32.0 % among 35–44 year-olds and 23.0 % among 65–74 year-olds Chinese [21]. Nevertheless, cleaning teeth at least once a day was nearly universal in this study (Table 1). Other studies have reported this figure to be quite low as 32.0 %[20]. Among those cleaning teeth, 88.2 % used toothbrush for cleaning their teeth in this study [11]. Variation in predisposing factors (oral health-related knowledge, attitudes, beliefs, perceptions and values), enabling factors (accessibility and availability of toothbrushes and toothpaste), together with different study sites, time, methodologies and nature of samples may account for this difference. Moreover, it could be explained as a lack of awareness among Nepalese population, as this picture was lowest among the non-formal education holders (Table 1). Nepal is a developing country with lower literacy rate [22] and higher poverty [23] which might have some linkage with the teeth cleaning habits. This is further supported by other findings in our study which revealed that odds of cleaning teeth twice a day and use of fluoridated toothpaste rose with a rise in educational level (Table 2). Previous studies have also documented more chances of teeth cleaning habit among educated people [4, 7, 20].

Women had higher odds of brushing teeth twice a day than men (Table 2), parallel to the conclusion of prior studies [4, 7]. Good oral hygiene behavior among women has been reported in various literatures [20, 24, 25]. The majority of the sampled women in the current study were homemakers (Table not shown), thus having flexible time. Being homemakers [26] and having flexible daily routine [27] have been suggested to promote higher teeth-cleaning behavior. Also, women are more motivated to follow oral hygiene practices, perhaps because of aesthetic value and more leisure time (major proportion of sampled women were housewives). Further evidence was illustrated by the current study with the higher odds of dental visit among the women (Table 2), and this goes in line with a previous study [28].

Fluoridated toothpaste use was common (71.4 %) in this study, higher than those documented by studies in Africa (18.0 %) [8] and China (5.0 %) [21]. Urban residents had twice greater odds of using fluoridated toothpaste than did the rural residents (Table 2) giving the idea that place of residence does matter in teeth cleaning habits, although the factors creating these differences might be the subject of further study. Sometimes, people in rural areas of Nepal clean their teeth with bamboo sticks, sacred twigs (*dattiwan*), Neem, charcoal, and salt in place of fluoridated toothpaste, which might not have been reported. Moreover, higher accessibility, availability, and variety of fluoridated toothpaste in urban areas of Nepal might have contributed to this variation. In line with an African study that found infrequent dental visits [7, 8], only four out of 100 respondents in this study reported visiting a dentist in the last 6 months. This contrasts with a western study that found nearly universal 6-monthly dental visits among the population [29], and with a Chinese study that reported a quarter of respondents making a dental visit in the last 12 months [21]. These differences may be explainable by oral health issues not drawing sufficient attention in Nepal, there being a lack of awareness on oral health issues, and a scarcity of dental health professionals [10]. Community education initiatives principally focusing on the importance of fluoride [30], fluoridated toothpaste and teeth cleaning twice a day as a means to fight oral health problems could be of importance [4, 17].

This study identified higher odds of dental visits in the last 6 months among the oldest age group, 45–69 years (Table 2), alike to the previous study with marked use of dental care among 45–64 years Swedish adults [6]. Logical assumption can be made that this age group might have more dental problems than the younger age group, as reportedly, burden of oral disease is concentrated more among the older people [31].

Like shown in earlier study [6, 28], the current study showed higher chances of dental visit among higher education holders than people with non-formal education. In comparison to rural residents, urban dwellers had two times higher odds of visiting a dentist within the last 6 months (Table 2). This could be argued to relate to the greater availability of sophisticated dental health services in urban areas of Nepal [10].

There is a strong need for the promotion of oral hygiene, as most of the Nepalese people cannot afford the costly treatments for dental diseases. Effective use of fluoride has been recommended as one of the important strategies to promote oral health at the population level [32]. Possibility of water fluoridation in Nepal is currently not feasible, with the lack of single supply system of water, rather salt fluoridation could be a possibility but has not started yet in Nepal [30].

Despite its robust design, this study suffered some limitations, such as a failure to provide evidence of causality for the socio-demographic correlates identified. Nevertheless, the large number of samples, representativeness of samples, and use of standardized tool ensured the reliability of the study.

Nonetheless, there is need for further studies in this domain - not only studies which draw subjective explanations for oral health conditions, but national oral health surveys using objective methods of examining oral health conditions.

## Conclusion

We found good oral hygiene practices especially cleaning teeth at least once a day and use of fluoridated toothpaste. However, cleaning teeth twice a day and visiting a dentist is less common. Being women, Terai residents, urban dwellers and educated were significantly associated with oral hygiene practices assessed in this study. Relevant stakeholders need to focus on promoting dental visits for dental care where feasible and making use of fluoridated toothpaste universal.

## Additional files

Additional file 1: Oral Hygiene Nepal Data. (XLSX 719 kb) Additional file 2: Code Book (STEPS Nepal 2013 Instruments). (PDF 340 kb)

## Abbreviations

AOR: Adjusted odds ratio
CI: Confidence interval
DHO: District Health Office
DPHO: District Public Health Office
ERB: Ethical Review Board
NCD: Non communicable disease
NHRC: Nepal Health Research Council
OR: Odds ratio
PDA: Personal digital assistant
PPS: Probability proportionate to size
PSU: Primary sampling unit
SPSS: Statistical package for the social sciences
SSU: Secondary sampling unit
TSU: Tertiary sampling unit
VDC: Village Development Committee
WHO: World Health Organization
